# Co-Occurrence Fingerprint Data-Based Heterogeneous Transfer Learning Framework for Indoor Positioning

**DOI:** 10.3390/s22239127

**Published:** 2022-11-24

**Authors:** Jian Huang, Haonan Si, Xiansheng Guo, Ke Zhong

**Affiliations:** 1 Department of Electronic Engineering, University of Electronic Science and Technology of China, Chengdu 611731, China; 2 Yangtze Delta Region Institute (Quzhou), University of Electronic Science and Technology of China, Quzhou 324000, China

**Keywords:** indoor positioning, heterogeneous transfer learning, co-occurrence data

## Abstract

Distribution discrepancy is an intrinsic challenge in existing fingerprint-based indoor positioning system(s) (FIPS) due to real-time environmental variations; thus, the positioning model needs to be reconstructed frequently based on newly collected training data. However, it is expensive or impossible to collect adequate training samples to reconstruct the fingerprint database. Fortunately, transfer learning has proven to be an effective solution to mitigate the distribution discrepancy, enabling us to update the positioning model using newly collected training data in real time. However, in practical applications, traditional transfer learning algorithms no longer act well to feature space heterogeneity caused by different types or holding postures of fingerprint collection devices (such as smartphones). Moreover, current heterogeneous transfer methods typically require enough accurately labeled samples in the target domain, which is practically expensive and even unavailable. Aiming to solve these problems, a heterogeneous transfer learning framework based on co-occurrence data (HTL-CD) is proposed for FIPS, which can realize higher positioning accuracy and robustness against environmental changes without reconstructing the fingerprint database repeatedly. Specifically, the source domain samples are mapped into the feature space in the target domain, then the marginal and conditional distributions of the source and target samples are aligned in order to minimize the distribution divergence caused by collection device heterogeneity and environmental changes. Moreover, the utilized co-occurrence fingerprint data enables us to calculate correlation coefficients between heterogeneous samples without accurately labeled target samples. Furthermore, by resorting to the adopted correlation restriction mechanism, more valuable knowledge will be transferred to the target domain if the source samples are related to the target ones, which remarkably relieves the “negative transfer" issue. Real-world experimental performance implies that, even without accurately labeled samples in the target domain, the proposed HTL-CD can obtain at least 17.15% smaller average localization errors (ALEs) than existing transfer learning-based positioning methods, which further validates the effectiveness and superiority of our algorithm.

## 1. Introduction

An indoor positioning system is a vital technique as it enables a series of position-based Internet of things (IoT) applications, including smart cities [1,2], healthcare [3], emergency rescue [4,5], occupancy prediction [6,7], and intelligent buildings [8,9,10]. Generally, accurate and real-time position data are required in the aforementioned GNSS-denied scenarios, which are usually hindered by complex indoor structures, real-time environmental changes, multipath interferences, and so on. Currently, there are four fundamental indoor positioning solutions for these issues, i.e., geometric-based methods [11,12], vision-based [13] methods, pedestrian dead reckoning (PDR) [14], and fingerprint-based methods [15,16]. Among them, the fingerprint-based method receives the most attention due to its flexible implementations and easy availability of fingerprint samples using smart devices. In typical fingerprint-based indoor positioning systems (FIPS) [15,16], base stations are deployed as access points (APs) that transmit signals to terminal fingerprint collection devices, such as Wi-Fi [17], Bluetooth [18], and ZigBee signals [19]. The so-called fingerprint denotes arbitrary signal features that can distinguish locations, such as the received signal strength (RSS) [2,20], channel impulse response (CIR) [21], and power delay Doppler profile (PDDP). Based on the fingerprints collected by the terminal devices, two positioning phases were constructed. One is the offline fingerprint database construction phase, where the fingerprints gained by terminal devices are stored in a fingerprint database as training samples. The other one is the online positioning phase, in which one compares the fingerprint from an unknown user’s position with the fingerprint database and develops a classifier to predict the user’s position. Conventional FIPS consist of two phases. Nevertheless, the complex indoor environment inevitably involves unexpected dynamics, multipath effects, and other interferences, resulting in distribution differences between the source and target domains, where conventional FIPS methods can no longer guarantee positioning accuracy. Meanwhile, fingerprint collection devices may differ in offline and online phases, and even a tiny variation in the holding posture of devices tends to lead to distinct features. Additionally, a long interval between offline and online phases may cause version or quantity changes in APs, further leading to unexpected cross-domain heterogeneities of the feature spaces. Therefore, knowing how to mitigate cross-domain feature space divergence and heterogeneity is critical for accurate and robust FIPS.

Transfer learning [22], for the purpose of improving learning results through transferring knowledge from one domain to a different related domain is a promising machine learning algorithm used to mitigate distribution divergence problems for FIPS [23]. In practical FIPS scenarios, both the feature space distribution and dimension heterogeneity exist, inspiring researchers to pay more attention to heterogeneously transferred learning methods.

In the community of heterogeneous transfer learning, in order to establish the connection between source and target domains with different feature dimensions, a small number of accurately labeled samples in the target domain is normally required, which contributes to semi-supervised learning [24,25]. In the field of FIPS—especially in the online positioning phase, and different from image and text classifications—obtaining accurately labeled samples is often labor-consuming, rendering existing semi-supervised heterogeneous transfer algorithms that are not practical for FIPS in the presence of the environment or collection device changes. Furthermore, regarding transfer learning, there is a long-lasting problem with how to determine if given source samples are valuable to the target domain classification task. In some cases where the source and target domains are not strongly related, blindly using domain adaptation methods may degenerate learning properties instead, which is usually called the “negative transfer” issue. To avoid such an issue, some coefficients evaluating the correlation between two samples were developed, including the Pearson correlation coefficient (PCC) [26], Gaussian kernel function [27], and bipartite graphs [28]. Unfortunately, most of them can only evaluate correlations between homogeneous domains, motivating the extension to more general heterogeneous scenarios.

Co-occurrence data are known for their ability to capture higher-order relationships between classes, concepts, and labels [29], which are usually employed to joint data from heterogeneous domains. Conventionally, the definition of co-occurrence data involves a moment when features of both source and target samples can be captured. For instance, a picture and its corresponding text description can be treated as an image–text co-occurrence sample, which is easy to capture in the computer vision community. In FIPS, both time and feature consistencies are rather difficult to maintain, which motivates us to extend such a definition to a relatively general-sense version.

Oriented by the aforementioned problems, a heterogeneous transfer learning framework based on co-occurrence data (HTL-CD) is proposed. First, the source domain data were mapped into the target feature space, enabling us to fulfill the positioning task in the same feature space. Within our framework, the marginal and conditional probability distributions between the source and target domains were narrowed down through a maximum mean discrepancy (MMD) restriction, ensuring a higher positioning precision. Meanwhile, co-occurrence data acted as the bridge linking the source and target domains, based on which the cross-domain correlation could be calculated even with different feature spaces. Subsequently, a topology correlation restriction was imposed on each sample, such that the mapped distance between two highly related samples was particularly small, in which case, more knowledge from the source domain was transferred to the target domain. Contrarily, little knowledge transfer took place between rarely related samples, avoiding the so-called “negative transfer” problem.

The main contributions of our proposed algorithm can be summarized as

- Within the HTL-CD framework, homogeneous knowledge transfer and knowledge transfer between heterogeneous domains can be attained. To cope with the heterogeneous problem caused by the collection device heterogeneity or real-time environmental changes, a projection from the source feature space to the target feature space was considered. In addition, by imposing a MMD restriction on the mapping matrix, the cross-domain marginal and conditional distributions were both aligned, giving rise to higher positioning accuracy and robustness in uncertain environment dynamics.
- The cross-domain correlation was evaluated with a correlation coefficient, which could precisely reflect whether and how the given source domain is effective in knowledge transferring to the target domain in both homogeneous and heterogeneous cases. Hence, it is possible to take full advantage of a fingerprint database and avoid the “negative transfer” issue.
- Through extending the current definition of co-occurrence data to a general-sense version, it was dramatically easier to collect co-occurrence fingerprint samples in FIPS. With the help of general-sense co-occurrence fingerprint data, joint sources, and target domains, the correlation between two arbitrary samples can be fairly evaluated, such that even in the existence of significant cross-domain divergence, an accurate and robust positioning performance can be achieved without resorting to common features or accurately labeled samples in the target domain.

The remainder of the paper is structured as follows. Related works are reviewed in Section 2 and a problem formulation is presented in Section 3. Then, a detailed deduction of the proposed HTL-CD is presented in Section 4. The experiment environment construction and results are presented in Section 5. Finally, the conclusions of our algorithm and potential future works are discussed in Section 6. Moreover, a list of abbreviations frequently used in this article is presented in Table 1.

## 2. Related Work

In this section, some related works are briefly reviewed, mainly concerning classical indoor positioning algorithms and transfer learning in FIPS.

### 2.1. Classical Indoor Positioning Algorithms

#### 2.1.1. Geometric-Based Methods

Geometric-based indoor positioning typically utilizes geometric principles (such as triangulation and trilateration to calculate positions via the measured range or direction information from reference points to a target. The time-of-arrival (TOA) in [30] collects the absolute travel time of the signal from a reference node (base station, beacon, access point, and so on) to the user’s equipment and computes the user’s localization by trilateration. The time-difference-of-arrival (TDOA) [31] adopts the travel time difference instead, avoiding the complex synchronization task. Notice that the measurement of the range as well as the angle heavily relies on the line-of-sight (LOS) conditions, which are hardly satisfied in practical indoor applications.

#### 2.1.2. Vision-Based Methods

Due to the rapid development of computational vision techniques [32], vision-based feature extraction techniques have exhibited remarkable improvements, enabling the acquisition of target positions through several images captured by static cameras located at known positions. In [33], the authors made a comprehensive review of existing computer vision-based indoor localization methods. In [34], the authors proposed a localization framework, where a convolutional neural network (CNN) was employed to observe the type and bounding box of the target; another neural network was employed for orientation and distance calculations. Moreover, simultaneous localization and mapping (SLAM) [35] has received more attention recently; it is capable of constructing maps, positioning, and detecting indoor static objects in real time. Unfortunately, these methods tend to be environmentally vulnerable and LOS conditions are normally required, which may hinder their applications in complex dynamic indoor scenarios. Moreover, the feature extraction accuracy is positively proportional to the computational burdens of neural networks, bringing about unexpected time delays in highly accurate positioning cases and, thus, real-time positioning tasks cannot be fulfilled.

#### 2.1.3. PDR

PDR [14] recursively updates the positioning results from the last iteration based on direction and distance data given by inertial sensors, which are completely self-dependent and can operate in both LOS and non-LOS environments. However, the accumulated errors resulting from inertial sensor accuracy constraints is a fatal drawback [36], and the initial heading direction and position are required every time, which heavily limits its real-world application.

#### 2.1.4. FIPS

FIPS are defined as signal features that are valuable for position classifications, such as fingerprints, RSS [2,37], and channel state information (CSI) [38]; as an example, conventional FIPS contain two phases, i.e., the offline fingerprint database construction phase and online positioning phase. In [39], by employing the fingerprint database as the training set, the maximum likelihood position of the target could be deduced through statistical inference. Moreover, refs. [40,41] computed the similarity between the fingerprint database and testing samples via Euclidean distance, cosine similarity, or other similarity matrices, and localized targets at the fingerprint location with the highest similarity. Additionally, ref. [42] incorporated knowledge distillation into CNN-based indoor positioning systems to distill knowledge from large deep CNNs into small CNNs; lower average positioning errors could be obtained via a simplified model. The teacher–assistant framework was deployed in [43] to allow for a simple CNN indoor positioning system, where knowledge from a large pre-trained network was transferred to a small network. Furthermore, due to the pervasive wireless infrastructures empowered by 802.11 Wi-Fi protocols, Wi-Fi-based FIPS [44,45] are easy-implemented in most indoor environments without any prior location information of Wi-Fi base stations. Wi-Fi base stations are mainly designed for information exchanges instead of localization, limiting localization accuracy. Moreover, in the offline and online phases of FIPS, different terminal devices, such as IOS and Android smartphones, as well as different holding postures, result in appreciable feature fluctuations and environmental changes because of pedestrian movements, furniture changes, and other indoor dynamics, further rendering the source and target domains with distribution and dimension heterogeneities, which are the major concerns of this paper.

### 2.2. Transfer Learning in FIPS

Transfer learning, as a different kind of machine learning method, concentrates on storing knowledge gained while solving one problem and applying it to a different (but related) problem [46]. Specifically, domain adaptation, as a classical method of transfer learning, exhibits remarkable properties in addressing cross-domain distribution differences, which is exactly one of the challenges we are aiming to conquer in FIPS. Typically, transfer component analysis (TCA) [47] and joint distribution adaptation (JDA) [48,49] are effective at narrowing down the distribution discrepancies between fingerprint databases and target samples, facilitating the subsequent high-accuracy positioning. In [50], a transfer learning-based approach was employed to learn common patterns in fingerprint data from different Wi-Fi RSS indoor positioning datasets, and smaller positioning errors were achieved. To take full advantage of finite training data, [51] used parameters of a pre-trained neural network trained with the data obtained from finite difference time domain simulations. Moreover, in most practical scenarios, real-time environmental changes and collection device variations tend to cause feature space diversity, inspiring researchers to develop heterogeneous transfer learning frameworks for FIPS. In [52], a common feature space was constructed by resorting to a cross-domain mapping, allowing to adopt domain adaptation methods for further knowledge transfer. Furthermore, [53] proposed a new feature extraction scheme by retaining only the most significant predictors; they selected the most efficient feature dimensions by utilizing a hybrid-based approach to reduce the training calibration efforts. However, the aforementioned works neglected a long-lasting problem on how to determine whether (and how) the source domain knowledge is valuable for the target positioning task. In other words, the correlation between source and target domains is hardly considered in FIPS; ideally, we want more knowledge transferred between more related domains, such that “negative transfer” can be eliminated. Some correlation metrics, such as PCC [26], Gaussian kernel function [27], and bipartite graphs [28] are meant to promote effective homogeneous knowledge transfer, while the correlation calculation between heterogeneous domains is still open for FIPS.

Co-occurrence data are known for their extraordinary abilities in jointing source and target samples even from heterogeneous domains, which provide potential solutions for us to solve feature space heterogeneity. In [54], by resorting to labeled source data and unlabeled auxiliary co-occurrence data, a hedge ensemble scheme was proposed to solve the online heterogeneous transfer issue. Moreover, Xu et al. [55] developed multiple spatial pairwise local co-occurrence descriptors to improve the resolutions of fingerprint images. Note that image-based co-occurrence data are easy to obtain, while in FIPS, the rigorous time and feature consistencies hinder the derivations of co-occurrence fingerprint samples, inspiring us to extend such definitions to general versions.

Oriented by the aforementioned problems, we propose the HTL-CD framework for FIPS, capable of narrowing down cross-domain distribution differences, capturing the degree of correlation in both homogeneous and heterogeneous cases, and facilitating better learning performances. Therefore, higher positioning precision and robustness against uncertain dynamics can be obtained for FIPS.

## 3. Problem Formulation

In the first place, we define the samples from the fingerprint database as the source domain data $D_{s}=\left\{X_{s},y_{s}\right\}={\left\{x_{s}^{i},y_{s}^{i}\right\}}_{i=1}^{n_{s}}$, where $x_{s}^{i}={\left[x_{s}^{i,1},x_{s}^{i,2},\ldots ,x_{s}^{i,m_{s}}\right]}^{T}$ is the *i*th fingerprint sample in the database, representing the feature of the *i*th position. $n_{s}$ and $m_{s}$, respectively, denotes the number of fingerprint samples and dimensions of each fingerprint. Moreover, $y_{s}^{i}$ is the actual label of *i*th fingerprint. Similarly, $D_{t}=\left\{X_{t}\right\}={\left\{x_{t}^{i}\right\}}_{i=1}^{n_{t}}$ are defined as the unlabeled target domain data to be positioned, in which $x_{t}^{i}={\left[x_{t}^{i,1},x_{t}^{i,2},\ldots ,x_{t}^{i,m_{t}}\right]}^{T}$ is the fingerprint of the *i*th test sample.

Traditionally, the key principle of FIPS is training a classifier Γ(·) based on the offline source data $D_{s}$, and utilizing $\Gamma (D_{t})$ to achieve positions of user targets. However, this is under the assumption of the same or similar marginal probability distributions between the source and target domains, i.e., $P\left(X_{s}\right)=P\left(X_{t}\right)$, which is unrealistic owing to real-time environmental changes. Additionally, because of the different types and holding postures of terminal devices, some sensors may not be detected in the online positioning phase, which means the source and target domains suffer from different feature dimensions and $m_{s}\neq m_{t}$. In such feature-missing cases, directly using traditional FIPS contrarily lead to remarkable positioning errors.

In this article, our objective was to design accurate and robust positioning FIPS using $D_{s}$ and $D_{t}$, in the existence of unexpected AP variations and environmental uncertainties.

## 4. Htl-Cd Framework

**Figure 1:**
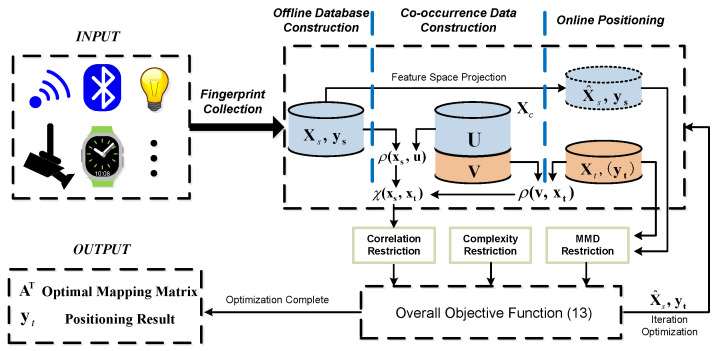
Overall architecture of the proposed HTL-CD.

In this section, the HTL-CD will be elaborated with the overall architecture illustrated in Figure 1. Evidently, it mainly consists of a MMD restriction, co-occurrence data construction, a correlation restriction, and optimization of the objective function.

### 4.1. MMD Restriction

Assume that there exists a mapping matrix $A\in R^{m_{s}\times m_{t}}$, such that the source data can be mapped into the target feature space through

(1) $${\hat{X}}_{s}=A^{T}X_{s}$$

The subsequent positioning is completely based on the mapped source data ${\hat{X}}_{s}=\left[{\hat{x}}_{1},{\hat{x}}_{2},\ldots ,{\hat{x}}_{n_{s}}\right]$, such that the selection of A is a crucial factor in the target positioning performance. Hence, the following MMD restriction is considered, with the purpose of adapting the mapped source data better to the target domain. Specifically, we hope that the marginal distribution of the mapped source data, as well as the conditional distribution, are aligned with those of the target data, such that the cross-domain distribution discrepancy can be narrowed down. Inspired by [48], the cross-domain marginal and conditional distribution distances can be measured by adopting the following empirical MMD:

(2) $$\begin{array}{cc} \mathrm{MMD} & \left({\hat{X}}_{s},X_{t}\right)=∥\frac{1}{n_{s}}\sum_{i=1}^{n_{s}}{\hat{x}}_{s,i}-\frac{1}{n_{t}}\sum_{i=1}^{n_{t}}x_{t,i}{∥}_{F}^{2}\\ \mathrm{MMD} & (y_{s}|{\hat{X}}_{s},y_{t}\left|X_{t}\right)=\sum_{c=1}^{C}∥\frac{1}{n_{s}^{\left(c\right)}}\sum_{i=1}^{n_{s}^{\left(c\right)}}{\hat{x}}_{s,i}^{\left(c\right)}-\frac{1}{n_{t}^{\left(c\right)}}\sum_{i=1}^{n_{t}^{\left(c\right)}}x_{t,i}^{\left(c\right)}{∥}_{F}^{2} \end{array}$$

with $n_{s}^{\left(c\right)}$ and $n_{t}^{\left(c\right)}$ notating the number of samples labeled with *c* in the source and target domain. *C* is the total number of different labels. Respectively, ${\hat{x}}_{s,i}^{\left(c\right)}$ and $x_{t,i}^{\left(c\right)}$ represent the *i*th sample with label *c* in the source and target domain.

For description conciseness, we denote $X=[{\hat{X}}_{s},X_{t}]$. Notice that $y_{t}$ in (2) cannot be obtained until the second iteration, inspiring us to consider the distribution in the following two cases:

  *Case 1*: In the first iteration, $y_{t}$ is unknown and the conditional distribution cannot be derived, such that only the marginal distribution is aligned and based on Equation (2), the distribution restriction is described as follows:

(3) $$\min_{A}\mathrm{MMD}\left({\hat{X}}_{s},X_{t}\right)=\min_{A}\mathrm{Tr}\left(X\left[\begin{array}{cc} \frac{1}{n_{s}^{2}}1_{s}1_{s}^{T} & -\frac{1}{n_{s}n_{t}}1_{s}1_{t}^{T}\\ -\frac{1}{n_{t}n_{s}}1_{t}1_{s}^{T} & \frac{1}{n_{t}^{2}}1_{t}1_{t}^{T} \end{array}\right]X^{T}\right)$$

*Case 2*: During the subsequent iterations, pseudo-labels for target samples were achieved via the last iteration, enabling us to minimize both marginal and conditional distribution differences, with the objective function expressed in the form of

(4) $$\min_{A}\left[\mathrm{MMD}\left({\hat{X}}_{s},X_{t}\right)+\mathrm{MMD}(y_{s}|{\hat{X}}_{s},y_{t}\left|X_{t}\right)\right]=\min_{A}\mathrm{Tr}\left({\mathrm{XMX}}^{T}\right)=\min_{A}\mathrm{Tr}\left(X\left[\begin{array}{cc} M_{s} & M_{st}\\ M_{ts} & M_{t} \end{array}\right]X^{T}\right)$$

with the detailed derivations of Equations (3) and (4) presented in Appendix A, and M contains

(5) $$\begin{array}{cc} \; & M_{s}=L_{s}+\sum_{c=1}^{C}L_{s}^{\left(c\right)},\;L_{s}=\frac{1}{n_{s}^{2}}1_{s}1_{s}^{T},\;{\left(L_{s}^{\left(c\right)}\right)}_{ij}=\left\{\begin{array}{cc} \frac{1}{{\left(n_{s}^{\left(c\right)}\right)}^{2}} & x_{i},x_{j}\in {\hat{X}}_{s}^{\left(c\right)}\\ 0 & \;\mathrm{others}\; \end{array}\right.\\ \; & M_{t}=L_{t}+\sum_{c=1}^{C}L_{t}^{\left(c\right)},\;L_{t}=\frac{1}{n_{t}^{2}}1_{t}1_{t}^{T},\;{\left(L_{t}^{\left(c\right)}\right)}_{ij}=\left\{\begin{array}{cc} \frac{1}{{\left(n_{t}^{\left(c\right)}\right)}^{2}} & x_{i},x_{j}\in X_{t}^{\left(c\right)}\\ 0 & \;\mathrm{others}\; \end{array}\right.\\ \; & M_{st}=L_{st}+\sum_{c=1}^{C}L_{st}^{\left(c\right)},\;L_{st}=-\frac{1}{n_{s}n_{t}}1_{s}1_{t}^{T},\;{\left(L_{st}^{\left(c\right)}\right)}_{ij}=\left\{\begin{array}{cc} -\frac{1}{n_{s}^{\left(c\right)}n_{t}^{\left(c\right)}} & x_{i}\in {\hat{X}}_{s}^{\left(c\right)},x_{j}\in X_{t}^{\left(c\right)}\\ 0 & \;\mathrm{others}\; \end{array}\right.\\ \; & M_{ts}=L_{ts}+\sum_{c=1}^{C}L_{ts}^{\left(c\right)},\;L_{ts}=-\frac{1}{n_{t}n_{s}}1_{t}1_{s}^{T},\;{\left(L_{ts}^{\left(c\right)}\right)}_{ij}=\left\{\begin{array}{cc} -\frac{1}{n_{t}^{\left(c\right)}n_{s}^{\left(c\right)}} & x_{i}\in X_{t}^{\left(c\right)},x_{j}\in {\hat{X}}_{s}^{\left(c\right)}\\ 0 & \;\mathrm{others}\; \end{array}\right. \end{array}$$

### 4.2. Co-Occurrence Data Definition

Co-occurrence data define a data set simultaneously containing source sample features as well as target sample features, which can be formulated as $D_{c}=\left\{X_{c}\right\}=\left\{\left[U;V\right]\right\}={\left\{\left[u_{p};v_{p}\right]\right\}}_{p=1}^{n_{c}}$, where U shares common features with $X_{s}$, V shares common features with $X_{t}$, and $n_{c}$ is the quantity of co-occurrence samples.

In the field of FIPS, heterogeneity is a rather complex problem, resulting from the heterogeneity of distributions, terminal device types, detected sensor numbers, and other environmental variations, such that there is no reasonable and specific definition for the co-occurrence data in heterogeneous FIPS. Borrowing the co-occurrence data [29] from the computer vision community, notated by narrow-sense co-occurrences for distinction, is presented as follows.

#### 4.2.1. Narrow-Sense Co-Occurrence Data

It is required that the collection devices, collection times, and labels of U and $X_{s}$, along with those of V and $X_{t}$, be strictly consistent.

Due to the different types and holding postures of terminal collection devices, some sensors may not be detected in the target positioning phases, hardly allowing us to seek a collection moment containing all of the involved APs. Consequently, collecting narrow-sense co-occurrence data is unpractical in the presence of real-time environmental changes, and a more general definition of co-occurrence data should be adopted.

#### 4.2.2. Wide-Sense Co-Occurrence Data

The rigorous device and time consistencies between U and $X_{s}$, V and $X_{t}$ are no longer required, such that the co-occurrence fingerprint data $X_{c}=\left[U;V\right]$ can be achieved through following the same trajectories in offline and target online phases and combining the collected fingerprints in both phases. For instance, a fingerprint sample $u_{i}$ is obtained at position $\mu_{i}$ in the offline phase, then we collect the fingerprint $v_{i}$ at the same position $\mu_{i}$ in the online positioning phase and a co-occurrence fingerprint sample $\eta_{i}=\left[u_{i};v_{i}\right]$ is derived. Repeating this process generates a group of co-occurrence fingerprint samples, capable of solving feature space heterogeneity.

In the sequel, unless declared otherwise, the so-called co-occurrence data refer to wide-sense co-occurrence data, which are much easier to obtain than the traditional narrow-sense co-occurrence data.

### 4.3. Correlation Restriction

Firstly, the correlation definition between two samples is divided into homogeneous and heterogeneous cases:

*Case 1*: Correlation coefficient of two homogeneous samples x,y is directly evaluated via PPC, calculated as

(6) $$\rho \left(x_{i},x_{j}\right)=\frac{\sum_{k=1}^{d}\left(x_{i}^{k}-{\bar{x}}_{i}\right)\left(x_{j}^{k}-{\bar{x}}_{j}\right)}{\epsilon +\sqrt{\sum_{k=1}^{d}{\left(x_{i}^{k}-{\bar{x}}_{i}\right)}^{2}}\sqrt{\sum_{k=1}^{d}{\left(x_{j}^{k}-{\bar{x}}_{j}\right)}^{2}}}$$

where $x_{i}=\left[x_{i}^{1},x_{i}^{2},\ldots ,x_{i}^{d}\right]\in R^{d}$ and $x_{j}=\left[x_{j}^{1},x_{j}^{2},\ldots ,x_{j}^{d}\right]\in R^{d}$. ${\bar{x}}_{i}=\frac{1}{d}\sum_{k=1}^{d}x_{i}^{k}$ and ${\bar{x}}_{j}=\frac{1}{d}\sum_{k=1}^{d}x_{j}^{k}$ respectively denotes the mean value of $x_{i}$ and $y_{j}$, with ϵ>0 being a small parameter.

Theoretically, the closer $\left|\rho (x_{i},x_{j})\right|$ is to 1, the more strongly related they are. $\rho (x_{i},x_{j})>0$ means $x_{i}$ and $x_{j}$ are positively correlated, and contrarily, $\rho (x_{i},x_{j})<0$ implies that $x_{i}$ and $y_{j}$ are negatively correlated.

*Case 2*: Correlation coefficient of two heterogeneous samples $x_{i},x_{j}$ is defined with the aid of $D_{c}=\left\{X_{c}\right\}=\left\{\left[U;V\right]\right\}={\left\{\left[u_{p};v_{p}\right]\right\}}_{p=1}^{n_{c}}$ in the following form:

(7) $$X\left(x_{i},x_{j}\right)=\max_{p=1}^{n_{c}}\rho \left(x_{i},u_{p}\right)\rho \left(x_{j},v_{p}\right)$$

where

(8) $$\begin{array}{cc} \; & \rho \left(x_{i},u_{p}\right)=\frac{\sum_{k=1}^{d_{x}}\left(x_{i}^{k}-{\bar{x}}_{i}\right)\left(u_{p}^{k}-{\bar{u}}_{p}\right)}{\epsilon +\sqrt{\sum_{k=1}^{d_{x}}{\left(x_{i}^{k}-{\bar{x}}_{i}\right)}^{2}}\sqrt{\sum_{k=1}^{d_{x}}{\left(u_{p}^{k}-{\bar{u}}_{p}\right)}^{2}}}\\ \; & \rho \left(x_{j},v_{p}\right)=\frac{\sum_{i=1}^{d_{y}}\left(x_{j}^{k}-{\bar{x}}_{j}\right)\left(v_{p}^{k}-{\bar{v}}_{p}\right)}{\epsilon +\sqrt{\sum_{i=1}^{d_{y}}{\left(x_{j}^{k}-{\bar{x}}_{j}\right)}^{2}}\sqrt{\sum_{i=1}^{d_{y}}{\left(v_{p}^{k}-{\bar{v}}_{p}\right)}^{2}}} \end{array}$$

in which $x_{i}=\left[x_{i}^{1},x_{i}^{2},\ldots ,x_{i}^{d_{i}}\right]\in R^{d_{i}}$ and $x_{j}=\left[x_{j}^{1},x_{j}^{2},\ldots ,x_{j}^{d_{j}}\right]\in R^{d_{j}}$. $u_{p}=\left[u_{p}^{1},u_{p}^{2},\ldots ,u_{p}^{d_{i}}\right]\in R^{d_{i}}$ and $v_{p}=\left[v_{p}^{1},v_{p}^{2},\ldots ,v_{p}^{d_{j}}\right]\in R^{d_{j}}$ respectively shares the same dimension with $x_{i}$ and $x_{j}$.

In the field of transfer learning, there exists an inherent problem as to how effective the source domain data are in transferring knowledge to the target domain. Aimed at addressing this problem, the following constraint is imposed on A, which can be expressed as

(9) $$\min_{A}\sum_{x_{i}\in X_{s}}\sum_{x_{j}\in X_{s}}\rho \left(x_{i},x_{j}\right){∥{\hat{x}}_{i}-{\hat{x}}_{j}∥}_{F}^{2}+\sum_{x_{i}\in X_{s}}\sum_{x_{j}\in X_{t}}2X\left(x_{i},x_{j}\right){∥{\hat{x}}_{i}-x_{j}∥}_{F}^{2}+\sum_{x_{i}\in X_{t}}\sum_{x_{j}\in X_{t}}\rho \left(x_{i},x_{j}\right){∥x_{i}-x_{j}∥}_{F}^{2}$$

Evidently, by resorting to the topology constrain in (9), the higher the correlations $x_{i}$ and $x_{j}$ are, the closer they will be in the mapped feature space, which is the so-called correlation restriction.

Furthermore, (9) can be rewritten in the following compact form:

(10) $$\min_{A}\mathrm{Tr}\left(XNX^{T}\right)$$

with

(11) N=D−W

in which $W\left(i,j\right)=w\left(x_{i},x_{j}\right)$ is a correlation matrix. $D=\mathrm{diag}\left(\sum_{j}W\left(:,j\right)\right)$ and W(:,j) represents the sum of the *j*th column elements of W.

### 4.4. Objective Function and Iterative Optimization

In order to restrict the complexity of matrix A and eliminate the overfitting issue, a regularization term is added along with (4) and (10), which generates the following overall objective function:

(12) $$\min_{A}\mathrm{Tr}\left(X\left(M+\alpha N\right)X^{T}\right)+\mathrm{Tr}\left(\beta A^{T}A\right)$$

with α and β being the design parameters, respectively, controlling the topology restriction and matrix complexities.

Let M+αN=H and $H=\left[\begin{array}{cc} H_{s} & H_{st}\\ H_{ts} & H_{t} \end{array}\right]$ and recalling that $X=[{\hat{X}}_{s},X_{t}]$ the final objective function F(·) is derived as

(13) $$\min_{A}F=\min_{A}\mathrm{Tr}\left(A^{T}X_{s}H_{s}X_{s}^{T}A+2A^{T}X_{s}H_{st}X_{t}^{T}\right.\left.+X_{t}H_{t}X_{t}^{T}+\beta A^{T}A\right)$$

Setting $\frac{\partial F}{\partial A}=0$ results in

(14) $$\frac{\partial F}{\partial A}=A^{T}X_{s}H_{s}X_{s}^{T}+X_{t}H_{ts}X_{s}^{T}+\beta A^{T}=0$$

Subsequently, the analytical expression of A in an extreme point is calculated in the following form:

(15) $$A^{T}=-\left(X_{t}H_{ts}X_{s}^{T}\right){\left(X_{s}H_{s}X_{s}^{T}+\beta I\right)}^{-1}$$

Employing ${\hat{X}}_{s}=A^{T}X_{s}$, the source domain data can finally be mapped into the feature space; in this space, traditional machine learning methods can be applied to construct a classifier for target positioning, e.g., K-nearest neighbor (KNN) [56] and support vector machine (SVM) [57]. A complete procedure of the proposed HTL-CD is presented in Algorithm 1.
**Algorithm 1:** HTL-CD framework for fingerprint positioning**Require:** $X_{s},y_{s}$, $X_{t}$, $X_{c}=\left[U,V\right]$, α, β, *T***Ensure:** $A^{T}$, ${\hat{y}}_{t}$  1:Compute N using (11);  2:Compute M using (3) and H=M+αN;  3:Compute $A^{T}$ using (15) and ${\hat{X}}_{s}=A^{T}X_{s}$;  4:Compute Γ(·) using $\left\{{\hat{X}}_{s},y_{s}\right\}$ and $y_{t}=\Gamma \left(X_{t}\right)$  5:**while** Iteration times ≤T **do**  6:       Compute M using (4) and update H=M+αN;  7:       Update $A^{T}$ using (15) and ${\hat{X}}_{s}=A^{T}X_{s}$;  8:       Update Γ(·) using $\left\{{\hat{X}}_{s},y_{s}\right\}$ and $y_{t}=\Gamma \left(X_{t}\right)$  9:**end while** 10:**return** $A^{T}$ and ${\hat{y}}_{t}$

## 5. Experiment Results

In this section, the positioning performance comparison between the proposed HTL-CD and existing FIPS are provided, along with the robustness analyses.

### 5.1. Environment Description

**Figure 2:**
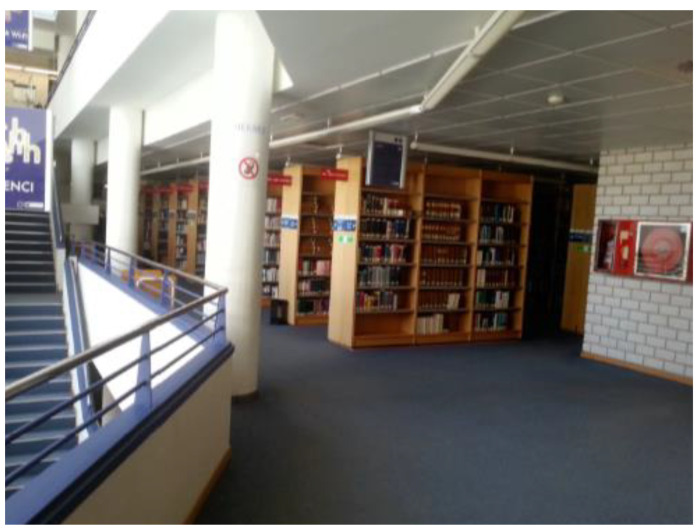
Experimental environment.

**Figure 3:**
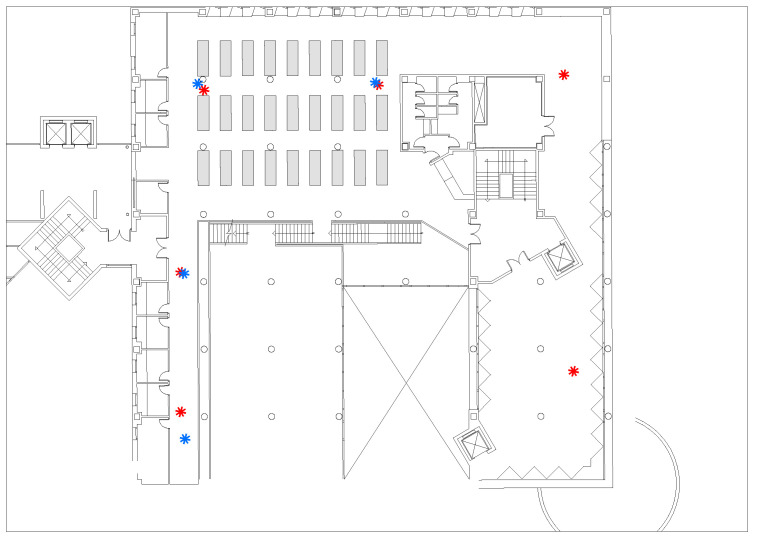
Layout of the experimental testbed.

In order to validate the robustness of our HTL-CD against the environmental changes, the experiment is conducted using UJI-DB [58], consisting of Wi-Fi RSS fingerprints collected for 25 consecutive months from the 3rd and 5th floors of Universitat Jaume I library. This environment is rather complex; it not only contains tables, chairs, benches, bookshelves, and other obstacles, it suffers from pedestrian movements, furniture changes, and other indoor dynamics, involving great challenges to our positioning tasks. Figure 2 presents an actual picture of the experiment environment, where 106 grids from 154.2 $m^{2}$ were respectively selected to collect fingerprint samples on the 3rd and 5th floors. Moreover, Figure 3 depicts the layout and device deployments of the experimental testbed, where the red asterisks represent the third floor’s devices, and the blue asterisks represent the fifth floor’s devices. In total, there were 620 Wi-Fi APs with unknown localizations.

**Figure 4:**
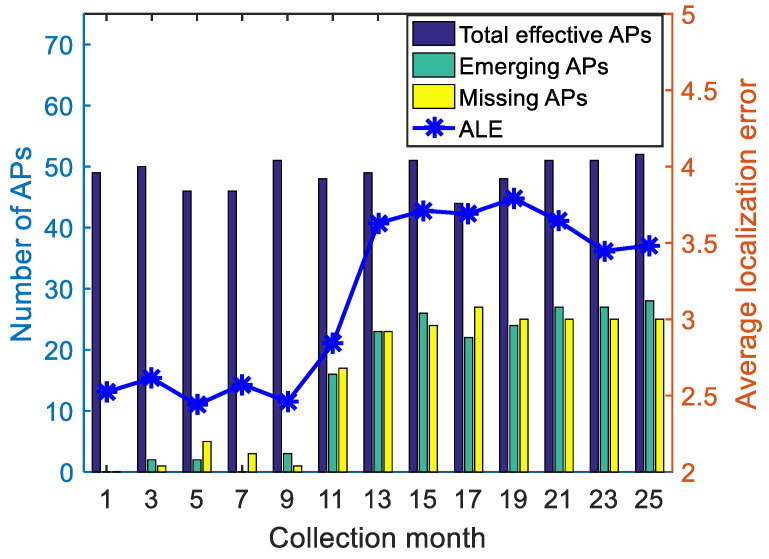
AP variations during the 25 months.

We set the fingerprint samples from the 1st month as the source domain and samples from other months as the target domain. In Figure 4, the number of effective APs and variations compared with the 1st month are depicted to reflect the sensor changes in 25 months. Emerging APs of the *i*th month define the APs detected in the *i*th month (and were not detected in the 1st month); the missing APs represent those detected in the 1st month (and were not detected in the *i*th month). Effective APs tremendously changed, especially after the 11th month, which severely deteriorated the positioning accuracies because of different feature spaces. Moreover, the number of the same APs from the 1st month and the average positioning errors directly using KNN are presented in Figure 4, implying that the positioning errors drastically increased as effective APs decreased, i.e., traditional classification schemes, such as KNN and SVM, could not guarantee positioning accuracy due to heterogeneous cross-domain feature spaces.

### 5.2. Positioning Results

The average localization error (ALE) was adopted to benchmark the accuracy capabilities for positioning algorithms, which can be formulated as

(16) $$E=\frac{1}{n_{t}}\sum_{i=1}^{n_{t}}||h\left({\hat{y}}_{t}^{i}\right)-h\left(y_{t}^{i}\right){\left|\right|}_{2}$$

where h(·) is a function transforming original label into 2D Cartesian coordinates. ${\hat{y}}_{t}^{i}$ is the prediction value of the actual label $y_{t}^{i}$.

To thoroughly and fairly compare the positioning performance, the following two cases were considered to conduct positioning experiments using the proposed HTL-CD along with current state-of-the-art schemes, including JDA [48], balanced distribution adaptation (BDA) [59], heterogeneous daily living activity learning (HDLAL) [25], heterogeneous domain adaptation using manifold alignment (DAMA) [60], and KNN.

*Case 1: positioning with partly different features.*

This case is designed to simulate a scenario with some sensors updated in the online positioning phase, i.e., the source and target domains share partially different features. Utilizing the samples from the 1st month as the source domain, the samples from the 12–25th months were the target domains to conduct our experiment. In addition, 100 co-occurrence samples were randomly selected by combining a source sample and a target sample with the same but unknown labels.

**Figure 5:**
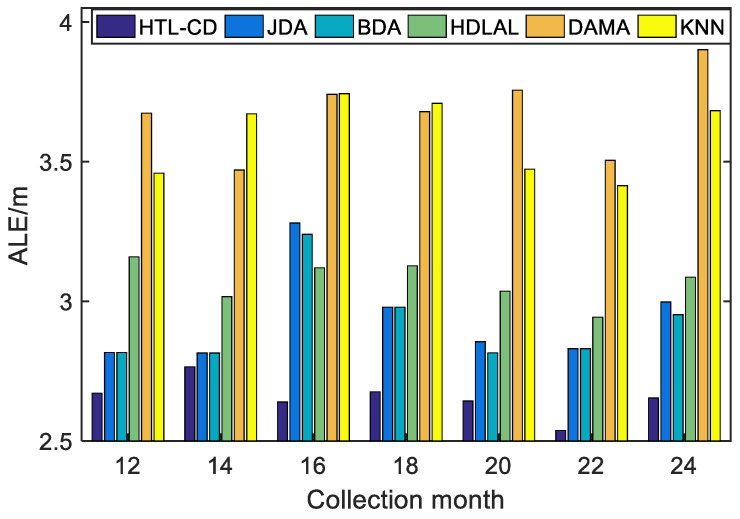
Comparison of the positioning performances with partially different APs.

The ALEs of these algorithms are depicted in Figure 5; apparently, HTL-CD exhibited more accurate and robust positioning results. Heterogeneous methods (HDLAL and DAMA) performed even worse than the homogeneous JDA and BDA methods, mainly because they did not take full advantage of the common features. By contrast, although our algorithm did not rely on common features, it employed co-occurrence data to joint all the samples via a correlation restriction, promoting knowledge transfer between related samples, such that the “negative transfer” issue was avoided and higher positioning precision and robustness could be gained.

*Case 2: positioning with totally different features.*

As a further illustration, an extreme circumstance was taken into account, where all the sensors were changed in the online positioning phase and the source and target domains owned totally different features. In accordance with case 1, with 2880 samples from the 1st month set as the source domain samples, and the samples from the 12th to 25th month being target domain samples, positioning tasks were fulfilled with the help of 100 randomly chosen co-occurrence samples.

**Figure 6:**
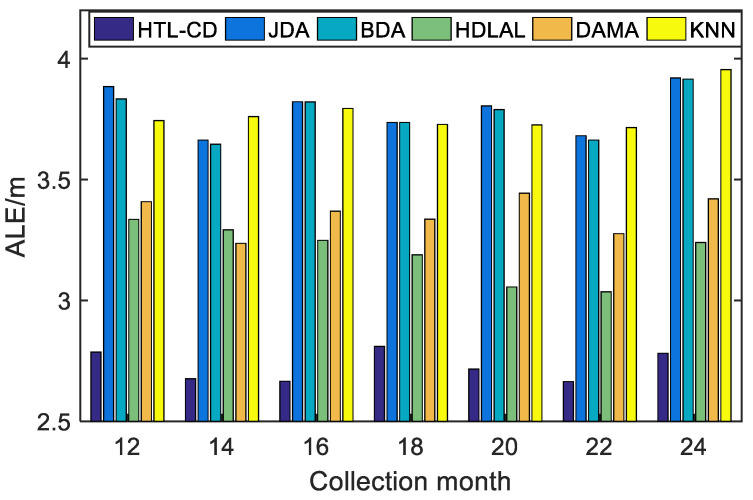
Comparison of the positioning performances with totally different APs.

The ALEs of our proposed HTL-CD along with state-of-the-art methods are presented in Figure 6; HTL-CD exhibits the most accurate and stable positioning properties. The source domain samples of homogeneous transfer methods mainly consisted of co-occurrence samples, thus they could not provide acceptable positioning precision due to their insufficient quantity, which can be observed in Figure 6. For comparison, heterogeneous methods obviously outperformed homogeneous ones, especially by resorting to co-occurrence samples, which relate the source domain samples with target domain samples; the proposed HTL-CD exhibits the highest positioning accuracy with ALE uniformly lower than 2.9 m, further demonstrating the superiority of the proposed position algorithm.

### 5.3. Robustness Analyses

**Figure 7:**
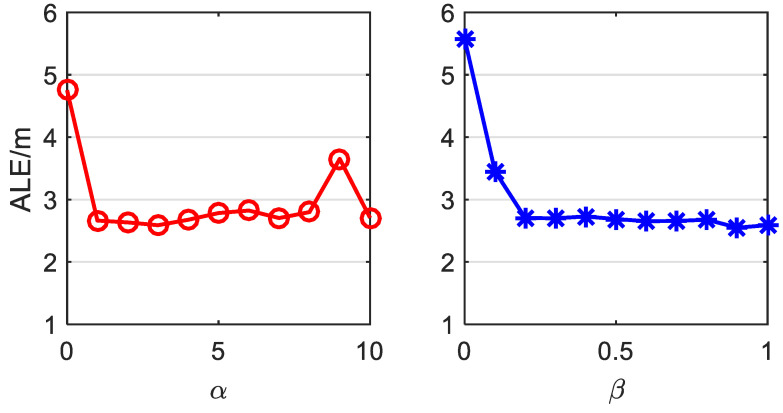
ALEs versus different α and β.

Notice that the design parameters α and β in (12) may affect the positioning results; this section is presented for the sensitivity analyses. Let α range from 0 to 10 and β from 0 to 1; the corresponding ALEs are plotted in Figure 7. This figure implies that as long as α and β are within a reasonable interval, i.e., 1≤α≤8 and 0.2≤β≤1, the ALE will fluctuate slightly within an acceptable range. Contrarily, if α or β is too small, the ALE will be much higher, which indicates that both correlation and complexity restrictions are effective in establishing accurate and robust FIPS. Hence, we can conclude that our proposed HTL-CD is not sensitive to parameters α and β, and high-accuracy positioning does not rely on specified values of design parameters.

**Figure 8:**
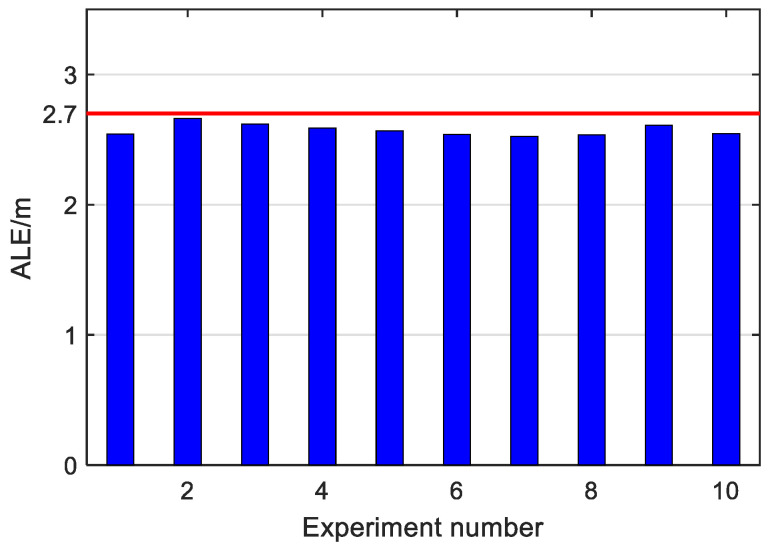
Experiments with random co-occurrence samples.

On the other hand, to study the dependence of the proposed HTL-CD upon specific co-occurrence samples, 10 experiments were further conducted, each with 100 randomly selected co-occurrence samples. The other design parameters were set in accordance with the last section. The ALEs of these experimental results are depicted in Figure 8. All positioning results were satisfactory with ALEs uniformly smaller than 2.7 m, which validates the effectiveness of our HTL-CD with random co-occurrence samples.

## 6. Conclusions

In this article, an HTL-CD framework is proposed, aimed at solving the time-varying distribution difference and feature space heterogeneity for FIPS, which can realize accurate and robust positioning performances without repeatedly reconstructing fingerprint databases. To solve the feature space heterogeneity issue, a cross-domain projection was considered, allowing us to design FIPS in a homogeneous space. Then, a MMD restriction was imposed on the projection; both marginal and conditional distribution differences could be minimized. Wide-sense co-occurrence data were used for joint sources and target domains, which are much easier to capture than traditional narrow-sense co-occurrence data in FIPS. Hence, correlation coefficients between both homogeneous and heterogeneous domains can be calculated and based on the employed correlation restriction; more knowledge transfer occurs between more correlated samples, which take full advantage of source samples and avoid the “negative transfer” issue. Subsequently, the MMD and correlation restriction are simultaneously embedded in an overall objective function; by iterative optimizations, optimal projection and positioning results can be obtained. An experimental performance implies that the proposed HTL-CD can obtain at least 17.15% smaller ALEs than existing transfer learning-based positioning methods, which further validates the effectiveness and superiority of our algorithm. Simultaneously, through robustness analyses, the positioning results of the proposed HTL-CD do not rely on specific design parameters or deliberately selected samples. Conclusively, the proposed HTL-CD can act well in long-term temporal variations in FIPS, while its application in different environments is still limited, which will be considered in future explorations. Moreover, the proposed HTL-CD scheme inevitably requires adequate samples in the source domain, which will motivate us to pay more attention to small-scale transfer FIPS in our future works.

## Figures and Tables

**Table 1:** Abbreviation list.

Notation	Meaning
$D_{s}$,$D_{t}$,$D_{c}$	source/target/co-occurrence domain
$X_{s}$,$X_{t}$,$X_{c}$	source/target/co-occurrence feature matrix
$y_{s}$,$y_{t}$	source/target labels
$n_{s}$,$n_{t}$,$n_{c}$	number of source/target/co-occurrence samples
$m_{s}$,$m_{t}$,$m_{c}$	dimension of source/target/co-occurrence samples
A,${\hat{X}}_{s}$	mapping matrix, mapped source feature matrix
N	correlation matrix
M	MMD matrix
K	kernelized matrix
$1_{s},1_{t}$	column vectors with $m_{s}$ and $m_{t}$ elements of value 1
α,β	topology and regularization parameters
*T*	iteration times
${∥⋆∥}_{F}$	Frobenius norm of ⋆
${\left(⋆\right)}^{T}$	transposition matrix of ⋆
Γ(⋆)	fingerprint classifier

## Appendix A

Detailed deductions of Equations (3) and (4) are given as follows:

(A1) $$\begin{array}{cc} \; & \min_{A}\mathrm{MMD}\left({\hat{X}}_{s},X_{t}\right)\\ = & \min_{A}∥\frac{1}{n_{s}}\sum_{i=1}^{n_{s}}{\hat{x}}_{s,i}-\frac{1}{n_{t}}\sum_{i=1}^{n_{t}}x_{t,i}{∥}_{F}^{2}\\ = & \min_{A}\mathrm{Tr}\left[\left(\frac{1}{n_{s}}\sum_{i=1}^{n_{s}}{\hat{x}}_{s,i}-\frac{1}{n_{t}}\sum_{i=1}^{n_{t}}x_{t,i}\right){\left(\frac{1}{n_{s}}\sum_{i=1}^{n_{s}}{\hat{x}}_{s,i}-\frac{1}{n_{t}}\sum_{i=1}^{n_{t}}x_{t,i}\right)}^{T}\right]\\ = & \min_{A}\mathrm{Tr}\left[\left(\frac{1}{n_{s}}\sum_{i=1}^{n_{s}}{\hat{x}}_{s,i}-\frac{1}{n_{t}}\sum_{i=1}^{n_{t}}x_{t,i}\right){\left(\frac{1}{n_{s}}\sum_{i=1}^{n_{s}}{\hat{x}}_{s,i}-\frac{1}{n_{t}}\sum_{i=1}^{n_{t}}x_{t,i}\right)}^{T}\right]\\ = & \min_{A}\mathrm{Tr}\left(\frac{1}{n_{s}^{2}}\sum_{i=1}^{n_{s}}\sum_{j=1}^{n_{s}}{\hat{x}}_{s,i}{\hat{x}}_{s,j}^{T}+\frac{1}{n_{t}^{2}}\sum_{i=1}^{n_{t}}\sum_{j=1}^{n_{t}}x_{t,i}x_{t,j}^{T}-\frac{1}{n_{s}n_{t}}\sum_{i=1}^{n_{s}}\sum_{j=1}^{n_{s}}{\hat{x}}_{s,i}x_{t,j}^{T}-\frac{1}{n_{s}n_{t}}\sum_{i=1}^{n_{s}}\sum_{j=1}^{n_{s}}x_{t,i}{\hat{x}}_{s,j}^{T}\right)\\ = & \min_{A}\mathrm{Tr}\left(\frac{1}{n_{s}^{2}}X_{s}\left(1_{s}1_{s}^{T}\right)X_{s}^{T}+\frac{1}{n_{t}^{2}}X_{s}\left(1_{t}1_{t}^{T}\right)X_{t}^{T}-\frac{1}{n_{s}n_{t}}X_{s}\left(1_{s}1_{t}^{T}\right)X_{t}^{T}-\frac{1}{n_{s}n_{t}}X_{t}\left(1_{t}1_{s}^{T}\right)X_{s}^{T}\right)\\ = & \min_{A}\mathrm{Tr}\left(X\left[\begin{array}{cc} \frac{1}{n_{s}^{2}}1_{s}1_{s}^{T} & -\frac{1}{n_{s}n_{t}}1_{s}1_{t}^{T}\\ -\frac{1}{n_{t}n_{s}}1_{t}1_{s}^{T} & \frac{1}{n_{t}^{2}}1_{t}1_{t}^{T} \end{array}\right]X^{T}\right) \end{array}$$

(A2) $$\begin{array}{cc} \; & \min_{A}\left[\mathrm{MMD}\left({\hat{X}}_{s},X_{t}\right)+\mathrm{MMD}(y_{s}|{\hat{X}}_{s},y_{t}\left|X_{t}\right)\right]\\ = & \min_{A}\left[\mathrm{MMD}\left({\hat{X}}_{s},X_{t}\right)+\sum_{c=1}^{C}\left(\frac{1}{n_{s}^{\left(c\right)}}\sum_{i=1}^{n_{s}^{\left(c\right)}}{\hat{x}}_{s,i}^{\left(c\right)}-\frac{1}{n_{t}^{\left(c\right)}}\sum_{i=1}^{n_{t}^{\left(c\right)}}x_{t,i}^{\left(c\right)}\right){\left(\frac{1}{n_{s}^{\left(c\right)}}\sum_{i=1}^{n_{s}^{\left(c\right)}}{\hat{x}}_{s,i}^{\left(c\right)}-\frac{1}{n_{t}^{\left(c\right)}}\sum_{i=1}^{n_{t}^{\left(c\right)}}x_{t,i}^{\left(c\right)}\right)}^{T}\right]\\ = & \min_{A}[\mathrm{MMD}\left({\hat{X}}_{s},X_{t}\right)+\sum_{c=1}^{C}(\frac{1}{{\left(n_{s}^{\left(c\right)}\right)}^{2}}\sum_{i=1}^{n_{s}^{\left(c\right)}}\sum_{j=1}^{n_{s}^{\left(c\right)}}{\hat{x}}_{s,i}^{\left(c\right)}{\left({\hat{x}}_{s,j}^{\left(c\right)}\right)}^{T}+\frac{1}{{\left(n_{t}^{\left(c\right)}\right)}^{2}}\sum_{i=1}^{n_{t}^{\left(c\right)}}\sum_{j=1}^{n_{t}^{\left(c\right)}}x_{t,i}^{\left(c\right)}{\left(x_{t,j}^{\left(c\right)}\right)}^{T}\\ \; & -\frac{1}{n_{s}^{\left(c\right)}n_{t}^{\left(c\right)}}\sum_{i=1}^{n_{t}^{\left(c\right)}}{\hat{x}}_{s,i}^{\left(c\right)}{\left(x_{t,j}^{\left(c\right)}\right)}^{T}-\frac{1}{n_{s}^{\left(c\right)}n_{t}^{\left(c\right)}}\sum_{i=1}^{n_{t}^{\left(c\right)}}x_{t,i}^{\left(c\right)}{\left({\hat{x}}_{s,j}^{\left(c\right)}\right)}^{T})]\\ = & \min_{A}[\mathrm{MMD}\left({\hat{X}}_{s},X_{t}\right)+\sum_{c=1}^{C}({\hat{X}}_{s}L_{s}^{\left(c\right)}{\hat{X}}_{s}^{T}+{\hat{X}}_{s}L_{st}^{\left(c\right)}X_{t}^{T}+X_{t}L_{ts}^{\left(c\right)}{\hat{X}}_{s}^{T}+X_{t}L_{t}^{\left(c\right)}X_{t}^{T})]\\ = & \min_{A}\mathrm{Tr}\left(X\left[\begin{array}{cc} M_{s} & M_{st}\\ M_{ts} & M_{t} \end{array}\right]X^{T}\right)\\ = & \min_{A}\mathrm{Tr}\left({\mathrm{XMX}}^{T}\right) \end{array}$$
